# *Salmonella* produce microRNA-like RNA fragment Sal-1 in the infected cells to facilitate intracellular survival

**DOI:** 10.1038/s41598-017-02669-1

**Published:** 2017-05-24

**Authors:** Hongwei Gu, Chihao Zhao, Tianfu Zhang, Hongwei Liang, Xiao-Ming Wang, Yi Pan, Xi Chen, Quan Zhao, Donghai Li, Fenyong Liu, Chen-Yu Zhang, Ke Zen

**Affiliations:** 1 State Key Laboratory of Pharmaceutical Biotechnology, Nanjing Advanced Institute for Life Sciences, School of Life Sciences, Nanjing University, Nanjing, Jiangsu 210046 China; 2 Jiangsu Engineering Research Center for MicroRNA Biology and Biotechnology, Nanjing, Jiangsu 210093 China; 3 School of Public Health, University of California at Berkeley, Berkeley, CA 94720 USA

## Abstract

*Salmonella* have developed a sophisticated machinery to evade immune clearance and promote survival in the infected cells. Previous studies were mostly focused on either bacteria itself or host cells, the interaction mechanism of host-pathogen awaits further exploration. In the present study, we show that *Salmonella* can exploit mammalian cell non-classical microRNA processing machinery to further process bacterial small non-coding RNAs into microRNA-like fragments. Sal-1, one such fragment with the highest copy number in the infected cells, is derived from *Salmonella* 5′-leader of the ribosomal RNA transcript and has a ‘stem’ structure-containing precursor. Processing of Sal-1 precursors to mature Sal-1 is dependent on host cell Argonaute 2 (AGO2) but not Dicer. Functionally, depleting cellular Sal-1 strongly renders the *Salmonella* bacteria less resistant to the host defenses both *in vitro* and *in vivo*. In conclusion, we demonstrate a novel strategy for *Salmonella* evading the host immune clearance, in which *Salmonella* produce microRNA-like functional RNA fragments to establish a microenvironment facilitating bacterial survival.

## Introduction

*Salmonella* spp. remains a leading cause of food-borne illness in the world. *Salmonella*, an enterobacteriaceae (rod-shaped, facultative intracellular parasites), infects both human and animals, each year causing approximately 1.3 billion cases of human diseases ranging from diarrhoea to typhoid fever^1^. Similar to other highly successful pathogens, *Salmonella* have developed various strategies to invade and survive in host cells^2–4^, including bypassing cellular anti-bacterial pathways^1^, subverting host innate and adaptive immune responses^2^, and eventually replicating inside the host cells^3, 4^. The strategies used by *Salmonella* are mainly dependent on bacterial components and generally fall into two categories: (a) active modification of its surface molecules (e.g., LPS, flagella, and peptidoglycan) to avoid host immune surveillance^2^, and (b) delivery of effectors into the eukaryotic host cells via the secretion systems (such as type III or type IV secretion system) to promote bacterial invasion, survival and replication inside the host cells^4–7^. After internalization, *Salmonella* can enter into the membrane-enclosed compartments (vacuoles and modified phagosomes) where they are protected from host defense mechanisms. In those compartments, *Salmonella* multiply freely before being released from the host cell to infect other cells. The ability to occupy such protected intracellular niche contributes to the pathogenesis of *S. enterica* ^8^.

It has been widely reported that unlike dead bacteria, living bacteria can release functional RNAs and proteins into the cytosol after they enter into the host cells^4, 9, 10^. Although the mechanism remains unclear, the delivery of bacterial materials into the cytosol of host cells is likely through active secretion or passive leakage out of the phagolysosome. In the cytosol, bacterial RNA can activate the host defenses. For example, Sander *et al*.^11^ showed that released bacterial RNA in the host cell cytosol bind to the receptors triggering a host response against the bacteria. Studying the mechanism underlying the vulnerability of *Listeria monocytogenes* to host defense, Schoen *et al*.^9^ showed that the released ovalbumin-encoding mRNA into the cytosol of epithelial cells, macrophages, and dendritic cells by that strain of *Listeria* was quickly processed and presented in MHC I molecules. In contrast to the ways in which bacterial mRNA can trigger host defenses against infecting bacteria, small non-coding RNA released by *Salmonella* and other pathogenic bacteria have recently emerged as mechanisms protecting the bacteria. These small non-coding RNA enable the bacteria to adapt to host environmental perturbations such as change in pH, osmotic pressure, as well as enabling intracellular replication^12, 13^. However, it remains unknown whether or how these small non-coding RNA can directly affect host cells by modulating mammalian cell gene expression in response to bacterial infection.

In the present study, we show that *Salmonella* can release small non-coding RNAs into the cytosol of the infected human intestinal epithelial cells, and within the host cell cytosol, these *Salmonella* RNA transcripts are further processed into ~22 nt miRNA-like functional RNA segments by AGO2-based non-classical miRNA processing machinery. One such RNA segment, Sal-1, has the highest copy number and can promote the intracellular survival of *Salmonella* in the infected cells. The present study thus identifies Sal-1 as a novel virulence factor for facilitating bacterial intracellular survival.

## Results

### Detection of *Salmonella*-encoded small RNA fragments in the infected mammalian cells

**Figure 1:**
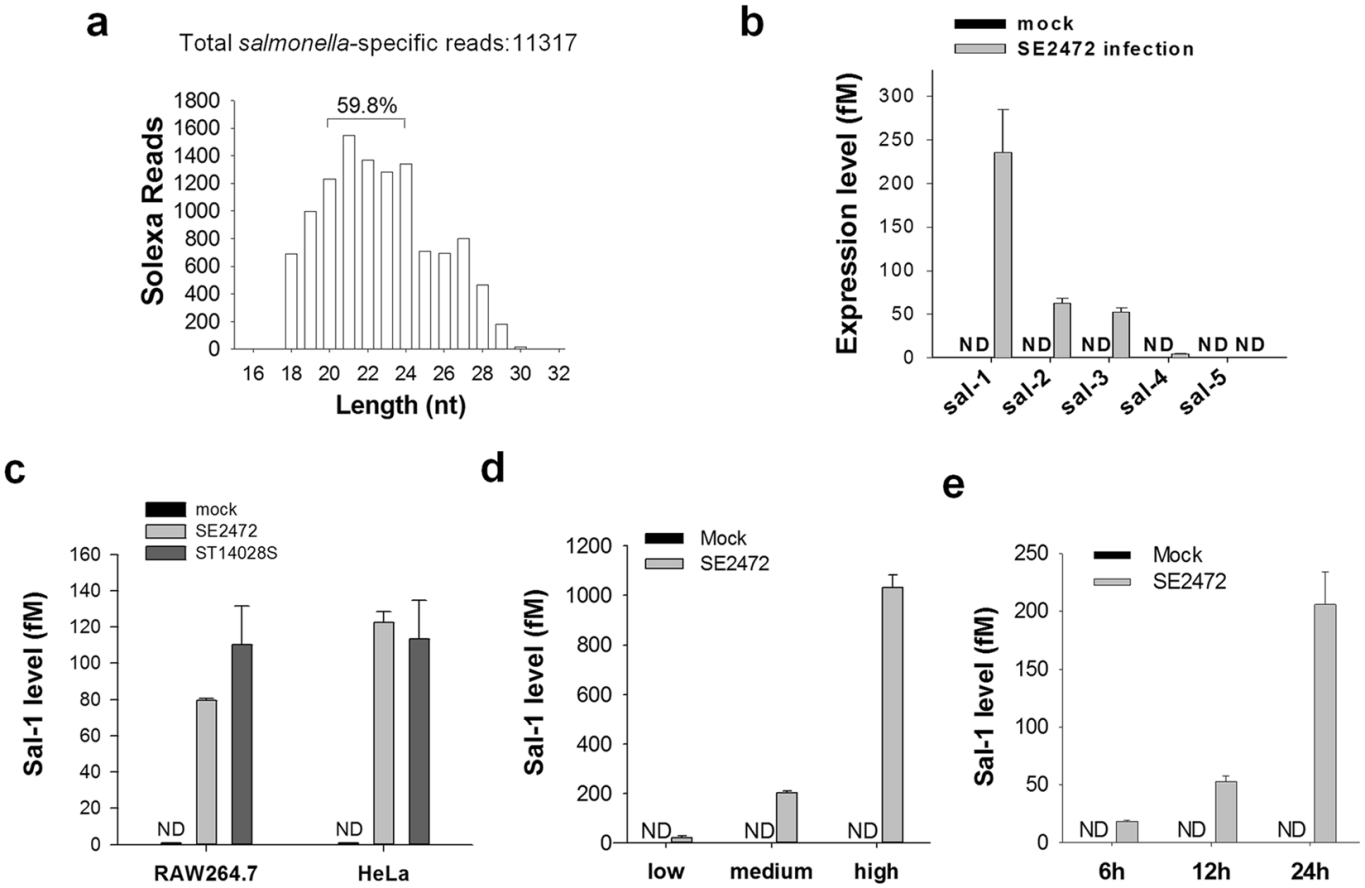
Production of small RNA fragments encoded by *Salmonella* in human intestinal epithelial cells infected by *Salmonella* strain SE2472. (**a**) Size distribution of *Salmonella*-encoded small RNA fragments. (**b**) Levels of five *Salmonella*-encoded small RNA fragments in HT-29 cells infected by SE2472. (**c**) Sal-1 level in RAW264.7 and HeLa cells infected with various *Salmonella* strains. (**d**) Sal-1 level in HT-29 cells at 12 h post-infection with SE2472 at MOI of 1 (low), 10 (medium) and 100 (high), respectively. (**e**) Sal-1 level in HT-29 cells at 6, 12 and 24 h post-infection with SE2472. The data are presented as the mean ± SEM (n = 4). ND, not detected.

Using the Solexa sequencing technique, we surveyed the global miRNA expression profile in intestinal epithelial HT-29 cells infected with *S*. *enteritidis* (strain SE2472). Cell lysate was centrifuged at high-speed and passed through a 0.22-μm filter to remove bacterial cells. In addition to a significantly altered expression profile of host cell endogenous miRNAs compared with that in the mock-infected cells, a panel of ~22-nt small RNA fragments derived from *Salmonella* were detected in *Salmonella*- infected cells (GEO accession number: *GSE53586*). The sequences and reads of these small RNA fragments (≥100 reads in Solexa sequencing) are listed in Supplemental Table S1. The copy number of Sal-1 was similar to that of endogenous miR-107. The sequences of these RNA segments did not match with the human genome but completely matched with *Salmonella* genome, suggesting that they were encoded by *Salmonella* and conserved in various S*almonella* strains. Interestingly, size analysis of these *Salmonella*-specific RNA fragments detected by Solexa sequencing showed a predominant distribution around 22 nt, a typical length of miRNA (Fig. 1a). The expression levels of five fragments (≥200 reads in Solexa sequencing) were further assayed by qRT-PCR with customised TaqMan probes. As shown in Fig. 1b, these fragments were detected in *Salmonella*-infected HT-29 cells but not in mock-infected cells. As the most enriched fragment, Sal-1 was assayed in other cell types infected with different *Salmonella* strains. Similar levels of Sal-1 were detected in HeLa and RAW264.7 cells infected with either SE2472 or *Salmonella* reference strain ST14028S (Fig. 1c). Production of Sal-1 in *Salmonella*-infected HT-29 was in a dose- and time-dependent manner (Fig. 1d and e), suggesting that more replication of bacteria in host cells yields more Sal-1. When HT-29 cells were infected with strain SE2472 at different multiplicities of infection (MOI of 1, 10 and 100) and assayed at 24 h post-infection, higher MOI of bacteria led to more bacteria invasion and more Sal-1 production (Fig. 1d). In HT-29 cells infected with strain SE2472 at MOI of 10, Sal-1 level was significantly increased from 6 h to 24 h post-infection (Fig. 1e).

**Figure 2:**
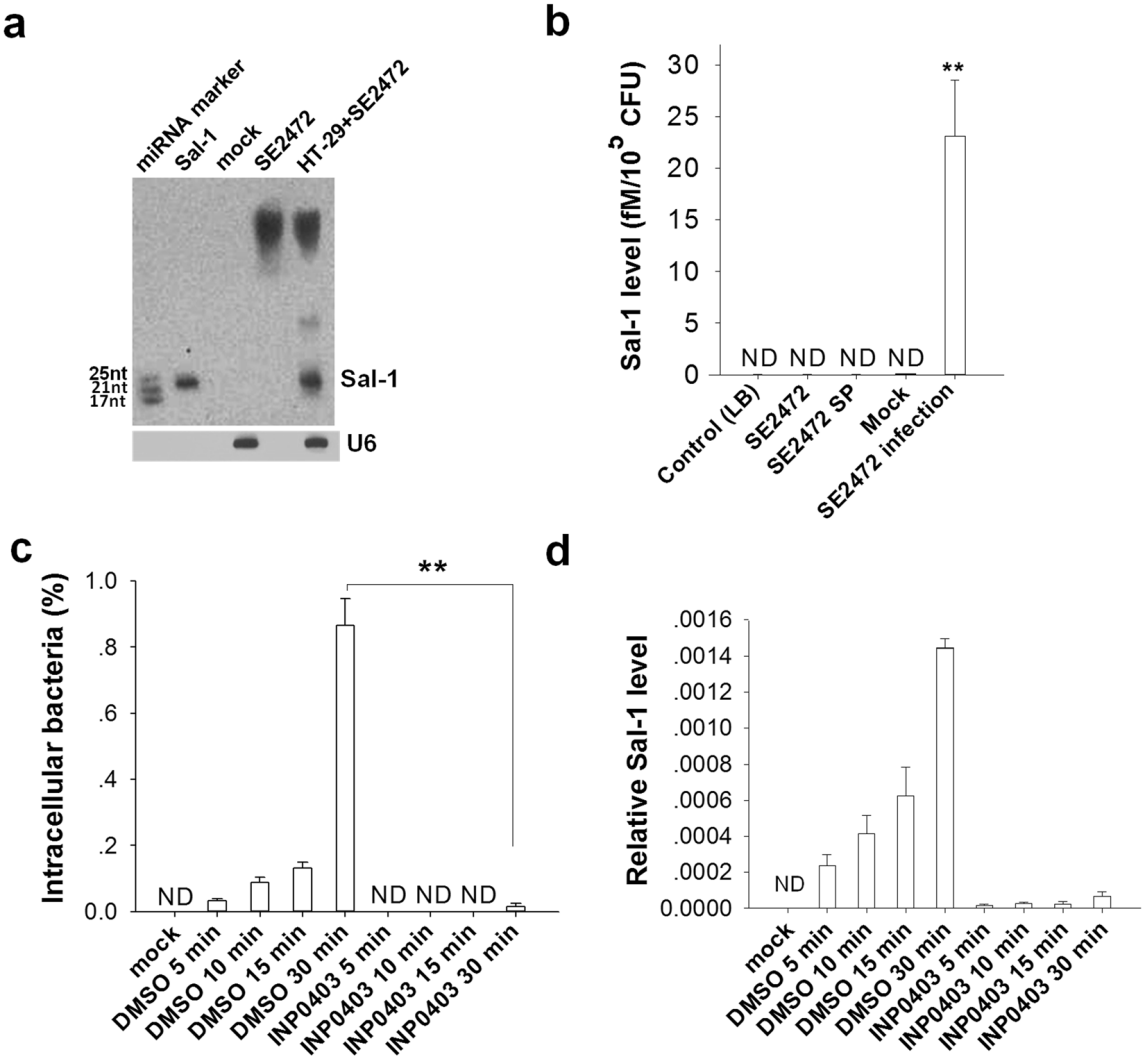
Production of mature Sal-1 in the *Salmonella*-infected intestinal epithelial cells. (**a**) Northern blot detection of Sal-1 in SE2472-infected HT-29 cells. (**b**) Sal-1 level in LB-grown SE2472, bacteria culture supernatant (SP) and HT-29 cells with Mock or SE2472 infection. (**c**-**d**) Bacteria number (**c**) and Sal-1 level (**d**) in the SE2472-infected HT-29 cells treated with or without T3SS blocker INP0403. The data are presented as the mean ± SEM (n = 4). ND, not detected. **P < 0.01.

The presence of Sal-1 in *Salmonella*-infected HT-29 cells was further confirmed by northern blot analysis (Fig. 2a) and qRT-PCR analysis (Fig. 2b), respectively. Interestingly, Sal-1 was only detected in samples of *Salmonella*-infected host cells (Fig. 2a). *Salmonella* alone (bacteria cultured in LB) only yielded large fragments of approximately 300 nt. In contrast, *Salmonella*-infected HT-29 cells generated not only large fragments but also medium and small fragments of ~70 nt and ~25 nt, respectively. The qRT-PCR analysis also confirmed that Sal-1 was only detected in *Salmonella*-infected HT-29 cells but not bacterial culture supernatant (SE2472 SP) (Fig. 2b), suggesting that Sal-1 must be processed in the host cells. This is consistent with previous report that bacteria themselves can only produce small non-coding RNAs of 50–300 nt but not of 20–24 nt^14^. In support of the observation that Sal-1 can only be generated in the host cells infected by *Salmonella*, we blocked the entry of strain SE2472 into HT-29 cells by INP0403, a small molecule that specifically inhibits T3SS^15^ and then tested the intracellular bacteria and Sal-1 level in HT-29 cells. As can be seen, compared with the DMSO vehicle control, INP0403 strongly blocked the entry of *Salmonella* SE2472 into the HT-29 cells (Fig. 2c), leading to no or little production of Sal-1 (Fig. 2d).

### Biogenesis of Sal-1 in the infected host cells

**Figure 3:**
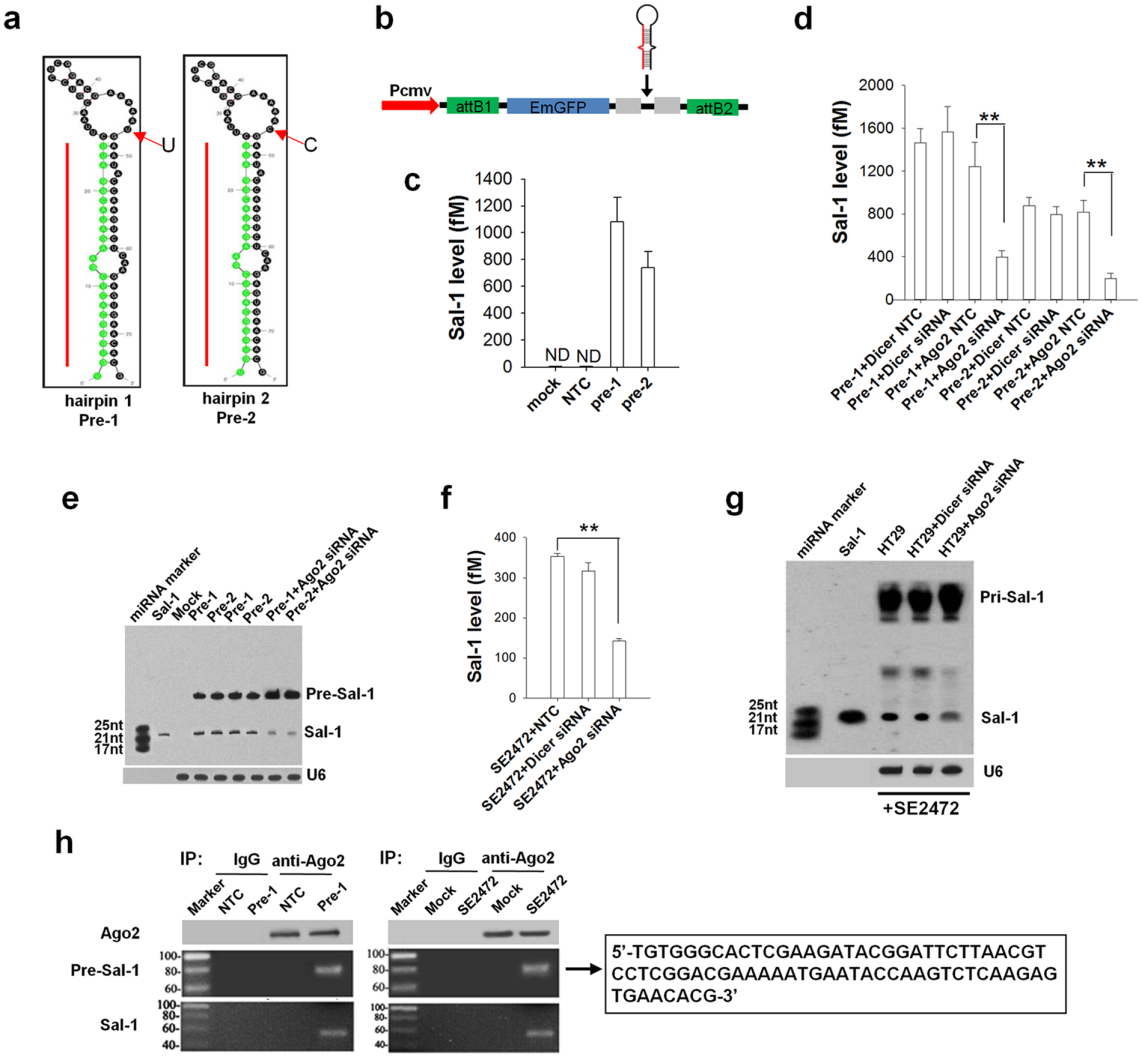
Biogenesis of Sal-1 in the *Salmonella*–infected HT-29 cells is AGO2-dependent. (**a**) Prediction of putative hairpin structure of Pre-Sal-1 for the biogenesis of mature Sal-1. (**b**) Construction of pcDNATM6.2-GW/EmGFP vector expressing Pre-Sal-1. Pre-Sal-1 was inserted immediately downstream of GFP and promoted by the CMV promoter. (**c**,**e**) Mature Sal-1 in HT-29 cells transfected with two Pre-Sal-1 expression plasmids (Pre-1 and Pre-2) detected by qRT-PCR **(c**) and northern blot analysis (**e**). (**d**) qRT-PCR detection of Sal-1 level in Pre-1- or Pre-2-transfected HT-29 cells with or without *Dicer* or *Ago2* silence. (**e**) Northern blot analysis of Sal-1 in Pre-1- or Pre-2-transfected HT-29 cells with or without *Ago2* silence. (**f**) qRT-PCR detection of Sal-1 level in SE2472-infected HT-29 cells with or without *Dicer* or *Ago2* silence. (**g**) Northern blot of Sal-1 and Pre-Sal-1 in SE2472-infected HT-29 cells with or without *Dicer* or *Ago2* silence. (**h**) RT-PCR amplification of Pre-Sal-1 and mature Sal-1 that are associated with AGO2 complex in HT-29 cells directly infected with SE2472 or transfected with Pre-Sal-1. Pre-Sal-1 was amplified, inserted into a T-vector and sequenced. The data are presented as the mean ± SEM (n = 3). **P < 0.01. ND, not detected.

We next characterized the biogenesis of Sal-1 in *Salmonella*-infected cells. As shown in Supplementary Fig. 1a and b, *Salmonella* genome (*S. enteritidis* P125109) contained seven copies of Sal-1, all of which were located at 92 nt upstream of the bacterial 16 S RNA gene in the non-coding region. Characterization of these seven Sal-1 copies (sites 1–7) was shown in Supplementary Fig. 2. The Sal-1 sequence was highly conserved among *Salmonella enterica spp*., *Escherichia coli*, and *Shigella spp*. The structure analysis showed that Sal-1 locus was 74 nt in length with two flanking arms of 24 nt for Sal-1 (left arm was marked with red frame), 26 nt for 3′ arm which is complementary to the ‘mature’ Sal-1 (marked with green frame), and a loop of 24 nt in between. This structure can be folded into a miRNA-like hairpin of Sal-1 ‘precursor’ (Pre-Sal-1). We thus speculated that mature Sal-1 (marked in red) could be produced through further digestion of two putative hairpin structures of Pre-Sal-1 (named Pre-1 for sites 1–3 and 5–7, and Pre-2 for site 4) (Fig. 3a). The sequence difference between Pre-1 and Pre-2 is located at the loop region (at position 47 within Pre-Sal-1 hairpin, “U” for Pre-1 and “C” for Pre-2). To confirm that hairpin structures of Pre-Sal-1 can be processed into mature Sal-1, the complete sequences of Pre-1 or Pre-2 were synthesized and inserted into the pcDNATM6.2-GW/EmGFP vector (Fig. 3b). Sal-1-expressing plasmids (Pre-1 and Pre-2) were then transfected into HT-29 cells, and the production of Sal-1 was analysed by qRT-PCR and northern blot analysis. As shown in Fig. 3c, Sal-1 was only detected by qRT-PCR in HT-29 cells transfected with Pre-1 or Pre-2, but not in non-transfected HT-29 cells or cells transfected with empty vector (negative control). Northern blot analysis of mature Sal-1 expression confirmed that Pre-Sal-1 can be properly processed into Sal-1 in host cells (Fig. 3e).

To test whether the generation of Sal-1 depends on its ‘stem-loop’ structure, we mutated Pre-1 at the 3′ arm to disrupt the sequence complementary to mature Sal-1. Although the sequence of mature (Mat) Sal-1 was not changed, the free energy of the mutated form of Pre-1 plasmid (Pre-1 Mut) was significantly higher (from ∆G = −25 kcal/mol to ∆G = −8.4 kcal/mol) (Supplementary Fig. 3a). After transfected into HT-29 cells, northern blot analysis showed that in contrast to Pre-1 WT-transfected cells in which Pre-Sal-1 was rapidly processed into Sal-1, cells transfected with Pre-1 Mut had an accumulation of Pre-Sal-1 but a strongly reduced Sal-1 production (Supplementary Fig. 3b).

### Maturation of Sal-1 in the *Salmonella*-infected cells is AGO2-dependent

Dicer is widely considered a central enzyme for cleaving short hairpins in classic miRNA processing^16^. However, for certain miRNAs such as miR-451, AGO2 is responsible for miRNA processing through a non-classical pathway^17, 18^. To determine whether the biogenesis of Sal-1 follows the processing pathways similar to miRNA, we knocked down Dicer or AGO2 in HT-29 cells via *Dicer*- or *Ago2*-specific siRNA and then determined Sal-1 level in HT-29 cells that were transfected with Pre-1 or Pre-2. As shown in Supplementary Fig. 4a and b, levels of Dicer and AGO2 were significantly decreased by transfection with *Dicer* siRNA and *Ago2* siRNA, respectively. In agreement with previous reports^19, 20^, level of miR-451 was not affected by Dicer knockdown but was significantly decreased by AGO2 knockdown (Supplementary Fig. 4c). Similarly, when HT-29 cells were co-transfected with Pre-1 or Pre-2 plus *Dicer* siRNA or *Ago2* siRNA, Sal-1 level was significantly decreased by *Ago2* siRNA but not *Dicer* siRNA (Fig. 3d). Northern blot analysis also showed that Sal-1 level was decreased by AGO2 knockdown and the reduction of Sal-1 was correlated to an accumulation of Pre-1 (Fig. 3e). Together, these results suggest that host cell AGO2 plays a critical role in the biogenesis of Sal-1.

AGO2-dependence of Sal-1 biogenesis was further confirmed in *Salmonella*-infected HT-29 cells. As shown in Fig. 3f, Sal-1 level in HT-29 cells infected with strain SE2472 was significantly reduced by AGO2 knockdown but not Dicer knockdown. Northern blot analysis of *Salmonella*-infected HT-29 cells treated with control siRNA or *Dicer* siRNA displayed three bands of 200–400 nt, ~70 nt and ~25 nt, likely the ‘primary’ form of Sal-1 (Pri-Sal-1), Pre-Sal-1 and mature Sal-1, respectively, whereas in *Salmonella*-infected HT-29 cells treated with *Ago2* siRNA, both the medium (~70 nt) and small (~25 nt) bands were significantly downregulated (Fig. 3g). The sequencing validation after amplification and insertion into T-vector confirmed that ~70 nt and ~25 nt bands were indeed the Sal-1 precursor (Pre-Sal-1) and mature Sal-1, respectively (data not shown).

To confirm the association of Sal-1 precursor or mature Sal-1 with AGO2 complex, we used anti-AGO2 antibody to immunoprecipitate AGO2 in HT-29 cells that were transfected with Pre-Sal-1 or infected directly with strain SE2472 (Fig. 3h), and then detected Pre-Sal-1 or mature Sal-1 in the immuneprecipitated complex by anti-AGO2 antibody. The results showed that both Pre-Sal-1 and mature Sal-1 were associated with the immunoprecipitated AGO2 complex. The sequence of the ~70 nt band collected from strain SE2472-infected cells completely matched that of Pre-Sal-1 (Pre-1).

### Procedure of process from Pri-Sal-1 to mature Sal-1

**Figure 4:**
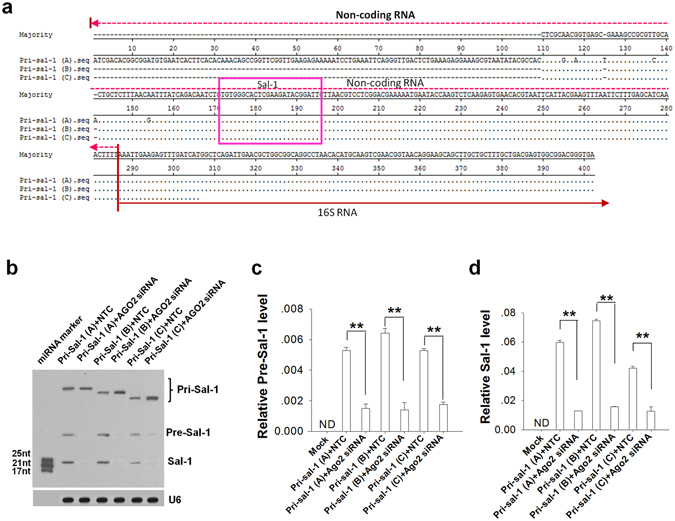
Identification of ‘primary’ form of Sal-1 in SE2472-infected HT-29 cells, which can be processed into pre-Sal-1 and mature Sal-1 sequentially. (**a**) Analysis of the sequences of three primary Sal-1 sites consisting of non-coding RNA and a partial region of the 5′ terminus of the 16 sRNA. (**b**) AGO2-dependent of Sal-1 generation from 3 possible Pri-Sal-1 (A, B and C) in HT-29 cells. Pri-Sal-1 (A, B and C) were amplified from infected HT-29 cells with 3′- and 5′- RT-RACE cDNA amplification followed by insertion into a pGEM-11Zf(+) vector for *in vitro* transcription. These primary Sal-1 RNA transcription fragments were then transfected into HT-29 for pre-Sal-1 and Sal-1 production. (**c**-**d**) Levels of pre-Sal-1 (**c**) and Sal-1 (**d**) in the pri-Sal-1-transfected HT-29 cells with or without silencing *Ago2*. The data are presented as the mean ± SEM (n = 3). **P < 0.01. ND, not detected.

To explore the source of Pri-Sal-1 in *Salmonella*-infected HT-29 cells, we performed 3′- and 5′- rapid amplification of cDNA ends (RT-RACE) to amplify primary Sal-1 in *Salmonella*-infected HT-29 cells (Supplementary Fig. 5). RNA was extracted from cell lysate that had passed through filter to remove the bacteria. As shown in Fig. 4a, we amplified three Pri-Sal-1 fragments, named Pri-Sal-1A (402 nt), 1B (291 nt), and 1 C (195 nt). These three Pri-Sal-1 fragments were all located in the non-coding region just upstream of the 16 S RNA gene, specifically at a distance of 92 nt from the 16 S RNA. The difference of three Pri-Sal-1 fragments in size (Pri-Sal-1B and Pri-Sal-1C) or sequence (Pri-Sal-1A) was likely due to their different location within *Salmonella* genome since Sal-1 could be transcribed from 7 different sites. The characterisation of Pri-Sal-1A, −1B and −1C in bacterial species was shown in Supplemental Tables S2–4. To confirm the AGO2-dependence of Sal-1 biogenesis, all three Pri-Sal-1 fragments were cloned into the pGEM-11Zf(+) vector, digested at the 3′-end, and transcribed by T7 RNA polymerase into primary Sal-1 RNA that were then transfected into HT-29 cells. As shown by northern blot (Fig. 4b) and qRT-PCR analysis (Fig. 4c and d), all the primary Sal-1 transcription fragments were successfully digested into Pre-Sal-1 and mature Sal-1, respectively, whereas the expression levels of Pre-Sal-1 (Fig. 4c) and mature Sal-1 (Fig. 4d) were significantly decreased when the cells were co-transfected with *Ago2* siRNA.

### Sal-1 increases the intracellular survival rate of *Salmonella*

**Figure 5:**
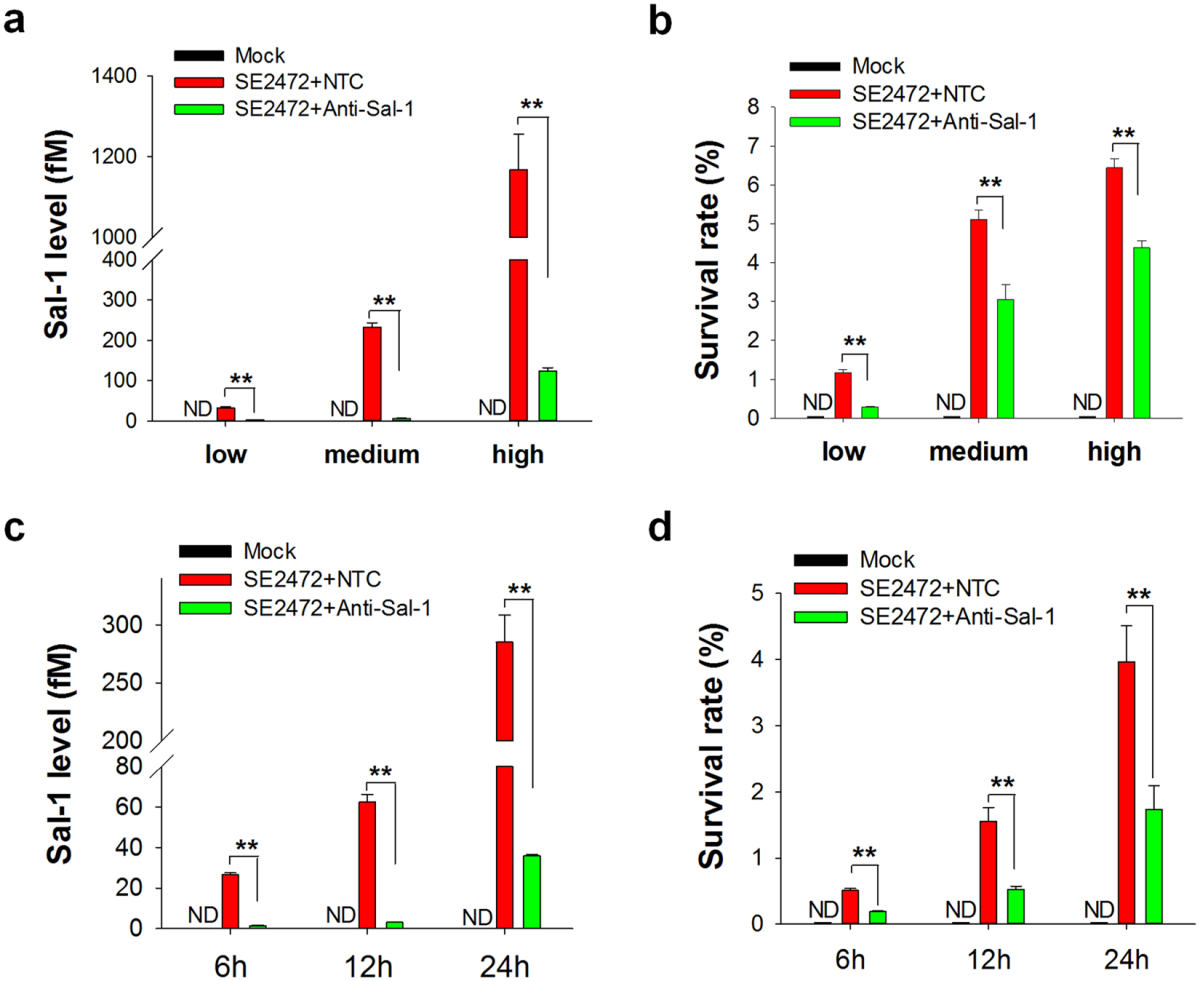
Sal-1 facilitates the intracellular bacterial survival following Salmonella infection. **(a**) Depletion of cellular Sal-1 by anti-Sal-1 oligonucleotide in HT-29 cells infected with SE2472 at MOI of 1, 10 and 100. (**b**) Bacterial survival rate in HT-29 cells infected with SE2472 at MOI of 1, 10 or 100. (**c**) Depletion of Sal-1 by anti-Sal-1 oligonucleotide in HT-29 cells infected with SE2472 for 6, 12 or 24 h. (**d**) Bacterial survival rate in HT-29 cells infected with SE2472 for 6, 12 or 24 h. Data are presented as the mean ± SEM (n = 3). ND, not detected. **P < 0.01.

Given the stable expression of Sal-1 in host cells and its positive correlation with *Salmonella* infection, we next explored the potential role of Sal-1 in the process of *Salmonella* infection. In the experiment, HT-29 cells were transfected with anti-Sal-1 oligonucleotide (Anti-Sal-1) to deplete cellular Sal-1 prior to *Salmonella* infection. As shown in Fig. 5a and c, the dose- or time-dependent increase of Sal-1 level in *Salmonella*-infected HT-29 cells, and Sal-1 was eliminated with anti-Sal-1 oligonucleotide. Depletion of cellular Sal-1 strongly reduced intracellular survival rate of strain SE2472 (Fig. 5b and d). These results collectively suggest that Sal-1 is involved in facilitating bacterial replication and survival in the infected cells.

**Figure 6:**
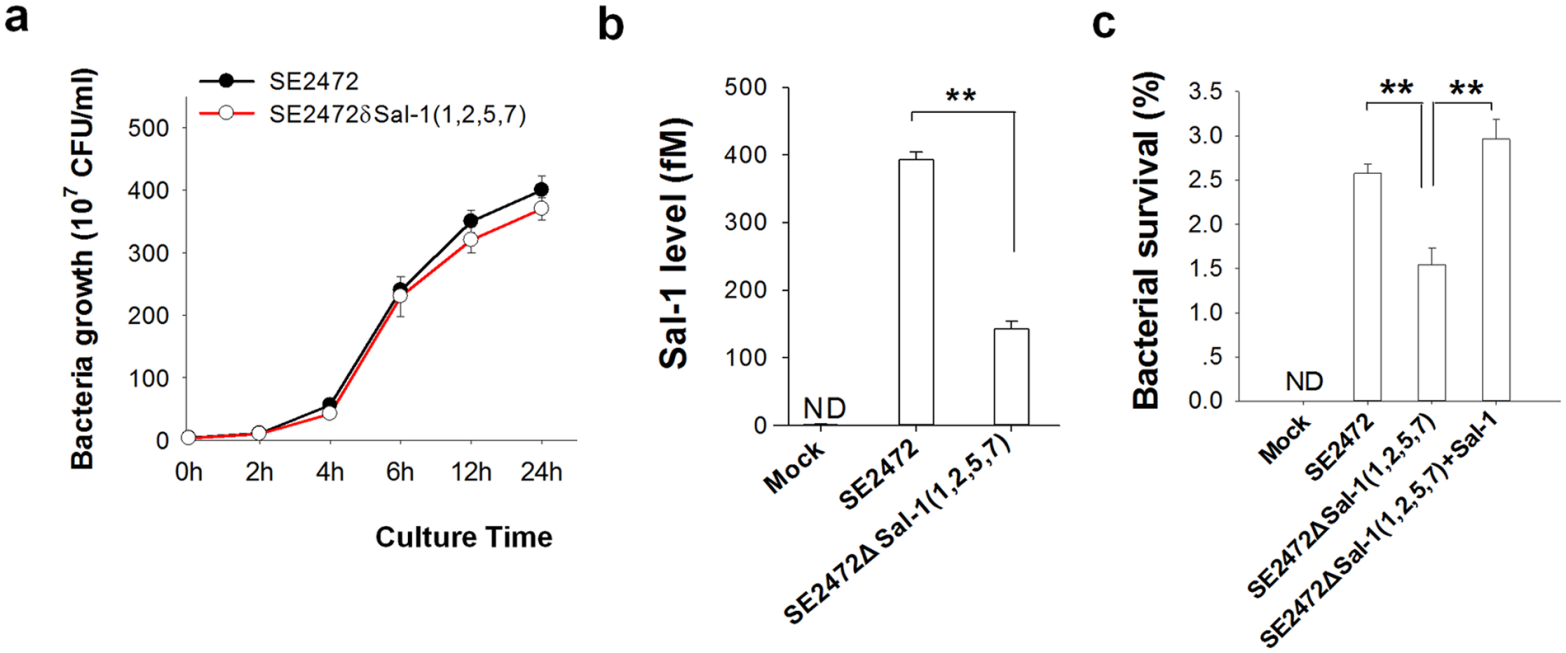
Deletion of Sal-1 sequence in bacterial genome decreases the bacterial intracellular survival rate following *Salmonella* infection. (**a**) Bacterial growth rate. (**b**) Level of Sal-1 in HT-29 cells infected with WT SE2472 or mutant SE2472∆Sal-1(1,2,5,7). (**c**) The level of bacterial intracellular survival rate in HT-29 cells infected with SE2472 or mutant SE2472∆Sal-1(1,2,5,7). Note that delivery of Sal-1 into SE2472∆Sal-1(1,2,5,7) -infected cells via Sal-1-expressing lentivirus largely repairs the defect of SE2472∆Sal-1(1,2,5,7) in promoting bacterial survival. ND, not detected. **P < 0.01.

To confirm the production of Sal-1 by *Salmonella*-infected cells and further test its role in bacterial intracellular survival, we employed “the Red Recombination System” to generate mutated SE2472 strain with Sal-1 sites being deleted. Given that deletion of all seven Sal-1 sites significantly impaired the bacterial growth, we generated a mutated SE2472 strain with only four Sal-1 sites being deleted, termed as SE2472∆Sal-1(1,2,5,7) (Supplementary Fig. 6). Strain SE2472∆Sal-1(1,2,5,7) displayed a similar growth curve in LB with strain SE2472 (Fig. 6a), suggesting that deletion of four Sal-1 sites in strain SE2472 does not affect bacterial growth. As expected, compared to WT strain SE2472, strain SE2472∆Sal-1(1,2,5,7) produced significantly less Sal-1 in HT-29 cells (Fig. 6b). Infection with strain SE2472∆Sal-1(1,2,5,7) resulted in a lower intracellular survival rate of bacteria compared to the WT SE2472 strain, and recovered with Sal-1(Fig. 6c).

### Sal-1 enhances *Salmonella* infection in mice

**Figure 7:**
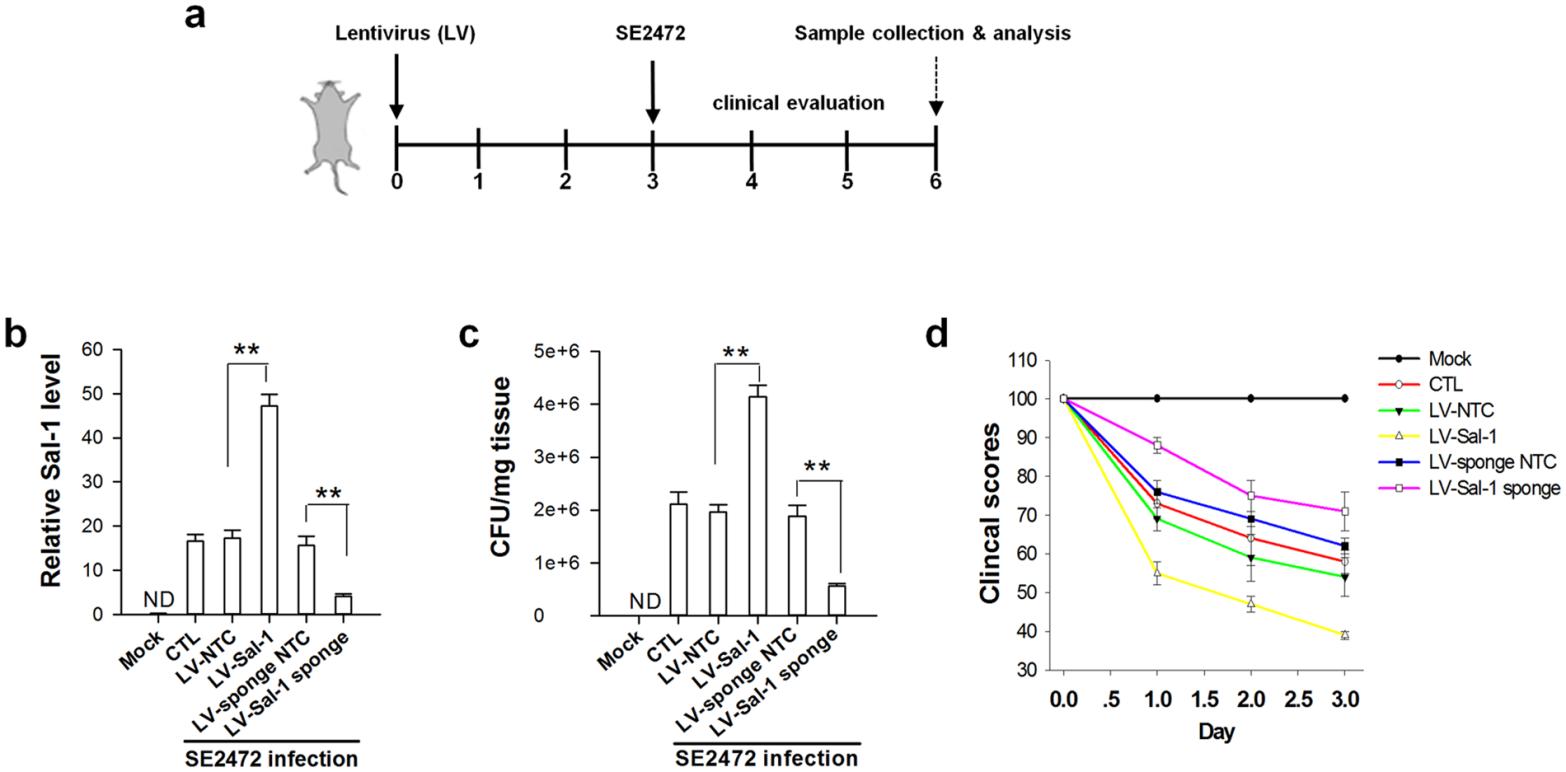
Role of Sal-1 in facilitating *Salmonella* infection in mice. The lentivirus expressing Sal-1 (LV-Sal-1) or Sal-1 sponge (LV-Sal-1 sponge) were constructed to overexpress or deplete Sal-1 in mouse colon epithelium. Prior to *Salmonella* infection, the lentiviruses were slowly administered into the lumen of mouse colon via a catheter. Female BALB/c mice (6–8 weeks) were then inoculated intragastrically with *Salmonella*. (**a**) Experimental design. (**b**) Sal-1 levels in mouse colon tissues. (**c**) *Salmonella* bacteria count in the mouse colon tissues. (**d**) The clinical scores of the mice after *Salmonella* infection. ND, not detected. **P < 0.01.

We next tested the role of Sal-1-targeting iNOS in *Salmonella* infection using mouse model. For this purpose, we constructed a Sal-1-expressing lentivirus vector (LV-Sal-1) to deliver Sal-1 in mouse colon tissues. In addition, a lentivirus vector containing three sequences that are completely complementary to Sal-1 was also constructed as a molecular sponge (LV-Sal-1 sponge) to absorb Sal-1. As depicted in Fig. 7a, mice were pre-treated with lentivirus vector (LV-Sal-1 or LV-Sal-1 sponge) or control lentivirus vector (LV-NTC). On the day 3, mice were then intragastrically infected with strain SE2472 (6 × 10^5^ CFU/ml). After euthanized mice on day 6, the mouse colon tissues were analysed for the levels of Sal-1 and bacterial intracellular survival. Sal-1 level in colon tissue was significantly elevated in *Salmonella*-infected mice compared with non-infected mice (Fig. 7b). The level of Sal-1 in *Salmonella*-infected colon tissues was further increased when the mice were treated with LV-Sal-1 but was markedly decreased when the mice were treated with LV-Sal-1 sponge. As an intracellular bacterial pathogen, infected *Salmonella* replicates in the intestinal epithelium. We thus determined the colonization levels of *Salmonella* in mouse colon tissues. As shown in Fig. 7c, colonies of *Salmonella* were detected in all the *Salmonella*-infected mice, whereas no *Salmonella* colonies were detected in the Mock infection group. The CFU counts in the strain SE2472-infected mice were significantly increased by LV-Sal-1 treatment but decreased by LV-Sal-1 sponge treatment. Together, these results suggest that increase of cellular level of Sal-1 promotes while depletion of cellular Sal-1 suppresses *Salmonella* intracellular survival.

A clinical scoring system, considering the factors, such as motility, ruffled fur, hunched position, feeding, ataxia and tremor, was further used to assess the systemic *Salmonella* infection in BALB/c mice. As shown in Fig. 7d, the mice in the mock infection group were completely healthy, with the highest score of 100. In contrast, the mice administered strain SE2472 or SE2472 plus control lentivirus vector (SE2472 + LV-NTC) displayed moderate illness and showed a clinical score of around 60. The mice administered strain SE2472 plus LV-Sal-1 (SE2472 + LV-Sal-1) developed a severe illness with the lowest clinical scores. The illness burden of *Salmonella* infection in mice, however, could be significantly alleviated by treatment with LV-Sal-1 sponge (SE2472 + LV-Sal-1 sponge) to deplete cellular Sal-1.

## Discussion

The concept that RNA molecules from viral or non-viral pathogens can act as regulators in eukaryotic cells has been around for some time. Early work showed that double-stranded RNA expressed by *E. coli* can silence genes in *C. elegans* ^21^. Subsequently, enteroinvasive *E. coli* were engineered to deliver regulatory short hairpin RNA (shRNA) molecules into mammalian cells^22^. Functional mRNAs delivered by engineered *Listeria* bacteria was shown to modulate the immune response of host cells^4^. Application of high-density tiling array and RNA sequencing technologies have recently shown that bacteria contain an extensive and previously unidentified repertoire of non-coding RNA, including 5′ and 3′ UTRs, antisense transcripts and intergenic small RNAs (sRNAs)^23, 24^. In addition to affecting various functional aspects of the bacteria, certain bacterial non-coding RNAs can also interfere with *C. elegans* genes and impact on the physiology of *C. elegans* ^25^. Recently Weiberg *et al*.^26^ showed that fungal “virulent” sRNA can target the gene of plant cells and suppress host immunity. During host-pathogen crosstalk, miRNA-mediated regulation has been recently reported^27^. *Samonella* engage miRNA to alter the host SUMOylome for raising its capacity for intracellular survival^28^. However, these RNAs mentioned above are all large-size RNA fragments, and it remains unknown whether small-size RNAs derived from pathogens have a similar posttranscriptional regulatory mechanism to control host gene expression. Against the traditional concept that bacteria cannot produce miRNA, here we demonstrate that, after entry into the host cells, pathogenic *Salmonella* can exploit host atypical miRNA processing machinery to further process its small non-coding RNAs into ~22 nt miRNA-like fragments, which in turn, directly regulate host cell gene expression at posttranscriptional level. This finding significantly expands the role of bacterial non-coding RNAs in modulating bacteria-host cell interplaying. Given that a panel of 19–24 nt milRNA fragments derived from *Salmonella* can be produced in the *Salmonella*-infected cells (Supplemental Table S1), production of such small functional RNA fragments might be a common phenomenon during enterobacteriaceae infection. As such ~22 nt *Salmonella* RNA fragments like Sal-1 can facilitate bacterial intracellular amplification and survival, they may represent novel virulence factors that are important for *Salmonella* infection and intracellular replication.

The endonuclease activity of AGO2 has been shown to play an important role in non-canonical maturation of miRNA^17–19, 29^. As a RNA-induced silencing complex (RISC) slicer, AGO2 plays a role in cleaving the pre-miRNA hairpin to an additional processing intermediate (termed as AGO2-cleaved precursor miRNA)^18^. Cleavage of pre-miR-451 by AGO2 is critical for maturation of miR-451^17^. Our result demonstrates that AGO2 actually mediates not only a single but two cleavage steps during maturation of bacterial Sal-1 in *Salmonella-*infected cells. As shown in Fig. 3, AGO2 is responsible for the cleavage of ‘pri-Sal-1′ (200–400 nt) to pre-Sal-1 (74 nt) and pre-Sal-1 to mature Sal-1 (24 nt). Knockdown of AGO2 resulted in an accumulation of ‘pri-Sal-1′ but less pre-Sal-1 and mature Sal-1 in *Salmonella-*infected cells. Our result suggests that, as an endonuclease, AGO2 might play an essential role in processing bacterial small RNA in the host cells. The capacity of AGO2 to cleave RNA fragments with various lengths may be due to a relative less strict restriction on the length RNA fragment required by AGO2, compared to Dicer.

Given that the production of Sal-1 is dependent on cellular AGO2 complex, bacteria alone cannot produce Sal-1. Therefore, although many non-pathogenic bacteria strains such as *E. coli* also contain Sal-1 sequence in their genome, mature Sal-1 will not be produced by the non-pathogenic bacteria strains and function as a virulence factor for them because these bacteria strains cannot enter into the host cells. For pathogenic enterobacteriaceae, it has been documented that large bacterial RNA fragments are released from bacteria into the host cells following the entry of bacteria into the cells^9, 10^. Although the mechanism remains unclear, the release of bacterial RNA fragments into the cytoplasm may be associated with active secretion and/or passive leakage out of the phagolysosome. Our results clearly showed that a 200–400 nt *Salmonella* RNA fragment, as the primary form of Sal-1, was released into the host cell cytoplasm in a time-dependent and dose-dependent manner. Inside the host cells, the bacterial Pri-Sal-1 was further processed by AGO2 into Pre-Sal-1 and Sal-1, accordingly.

In addition, we performed *Ago2* silence experiments to further explore the link between Ago2 maturation of Sal-1 and host sensitivity to *Salmonella* cells. As shown Supplementary Fig. 7, *Ago2* siRNA treatment decreased the intracellular survival of both two *Salmonella* strains. Also, Sal-1 mutant SE2472ΔSal-1(1, 2, 5,7) displayed a lower intracellular survival rate than WT SE2472 strain, and the intracellular survival rate of SE2472ΔSal-1(1, 2, 5,7) was further decreased by *Ago2* siRNA treatment. We speculated that AGO2 depletion affect many other events, including the all miRNA function in the host cells, so the factors other than Sal-1 may also contribute to the decrease of bacterial intracellular survival after *AGO2* depletion.

In the present study, we show that *Salmonella* can exploit mammalian cell AGO2-mediated non-classical miRNA processing machinery to further process their non-coding RNA fragment of ribosomal or transfer RNA transcript into ~22 nt milRNA fragments. Specifically, in the infected host cells, *Salmonella* can process the fragment of the 5′ leader of bacterial ribosomal RNA operon transcript into 24 nt Sal-1, which in turn, facilitates the intracellular amplification and survival of *Salmonella*. Thus, the milRNA fragments like Sal-1, produced by *Salmonella* in the infected cells, may represent a novel class of virulence factors associated with bacterial infection and intracellular survival, and its potential pathogenic mechanism waiting for further study.

## Materials and Methods

### Cells, reagents and antibodies

Human intestinal epithelial cells (HT-29) and Hela were purchased from the China Cell Culture Centre (Shanghai, China) and maintained at 37 °C in a humidified 5% CO_2_ incubator with RPMI-1640 medium (Gibco, Carlsbad, CA) containing 10% FBS (Gibco), 100 units/ml penicillin, and 100 μg/ml streptomycin. Human embryonic kidney (HEK-293T) and mouse RAW264.7 cells (ATCC) were grown in Dulbecco’s modified Eagle’s medium (DMEM) supplemented with 10% FBS. Cell transfection was performed using Lipofectamine™ 2000 (Invitrogen, Carlsbad, CA). The anti-AGO2 (ab57113 for IP, and ab32381 for WB), anti-Dicer (ab14601), Normal mouse IgG was purchased from Millipore (Cat. 12–371). Synthetic RNA molecules, including *Dicer* and *Ago2* siRNA, antagomir anti-Sal-1 (2′-OMe-modified, cholesterol-conjugated), and scrambled control oligonucleotides, were purchased from GenePharma (Shanghai, China). Oligonucleotide probes containing locked nucleic acids (LNA, Exiqon) used for the Northern blot and DIG non-radioactive nucleic acid labelling and detection were purchased from Roche.

### Bacteria strains and invasion assay

Highly virulent WT *Salmonella enteritidis* strain SE2472^30^ (the isolate with low LD50 (<10^3^ organisms) and mortality occurred rapidly) was grown in Luria-Bertani (LB) broth. *E. coli* strains Top 10 and DH5α were used as hosts to amplify the plasmids. The cell invasion assay was performed as described previously^30^. Briefly, for the infection, HT-29 or RAW264.7 cells were seeded in six-well cell culture plates (Costar, Corning, NY) in culture medium without antibiotics. The bacteria were inoculated in 2 ml of LB medium and incubated at 37 °C overnight without shaking. The bacterial suspensions were diluted to a multiplicity of infection (MOI) of approximately five to ten. The plates were centrifuged at 1,000 rpm for 5 min and incubated at 37 °C for 2 h. After extensive washing, cell culture medium with gentamicin (50 μg/ml) was added, and the plates were incubated for 1.5 h at 37 °C. Next, the cells were washed five times, culture medium with gentamicin (10 μg/ml) was added, and the plates were incubated for 6, 12 or 24 h. The cells were then washed and lysed in 1 ml of phosphate-buffered saline (PBS) with 0.1% Triton X-100. The lysates were pipetted vigorously to release the intracellular bacteria, which were removed by filtering through a 0.22-μm membrane. A ten-fold dilution series was performed for the lysates, and they were plated onto LB plates for incubation overnight at 37 °C. The number of CFUs on the plates were counted and compared with the number of input bacteria. The invasiveness of *Salmonella* was measured by determining the percentage of intracellular bacteria, which was calculated as: number of intracellular bacteria/number of input bacteria) × 100. For S anti-Sal-1 antisense oligonucleotide (ASO) treatment, cells were first transfected with anti-Sal-1 ASO via Lipofectamine 2000, then infected with *Salmonella* on the second day. After infection, cells were washed with PBS followed by treatment with medium containing 50 μg/mL gentamicin to kill extracellular bacteria for 1.5 h. Cells were continuously cultured in the medium containing 10 μg/mL gentamicin for 24 h, and then harvested for assessment of bacterial survival.

### RNA isolation and qRT-PCR

Total RNA was extracted from *Salmonella* strain SE2472, *Salmonella*-infected cells, or whole tissues using TRIzol reagent or TRIzol LS reagent (Invitrogen) according to the manufacturer’s instructions. Quantitative RT-PCR was performed using TaqMan Sal-1 probes (Applied Biosystems, Foster City, CA) according to the manufacturer’s instructions. Briefly, total RNA was reverse transcribed to cDNA using AMV reverse transcriptase (Takara) and a stem-loop RT primer (Applied Biosystems). Real-time PCR was performed using a TaqMan PCR kit and an Applied Biosystems 7300 Sequence Detection System (Applied Biosystems). All the reactions, including the no-template controls, were performed in triplicate. After the reaction, the C_T_ values were determined using fixed threshold settings. To calculate the absolute expression levels of the target Sal-1, a series of synthetic Sal-1 oligonucleotides at known concentrations was reverse transcribed and amplified. The absolute amount of each Sal-1 was calculated in reference to the standard curve, or the Sal-1 expression in the cells was normalised to the U6 snRNA level.

### Solexa sequencing

Solexa sequencing was performed as previously described^31^. In brief, total RNA was extracted from strain SE2472-infected HT-29 cells (which were lysed with PBS containing 0.1% Triton X-100 and filtered through a 0.22-μm membranes) using TRIzol LS reagent (Invitrogen, Carlsbad, CA). After polyacrylamide gel electrophoresis (PAGE) purification of the small RNA molecules (less than 30 base pairs) and ligation of a pair of Solexa adaptors to their 5′ and 3′ ends, the small RNA molecules were amplified using the adaptor primers for 17 cycles, and those fragments of approximately 90 bp (small RNA + adaptors) were isolated from the PAGE gels. The purified DNA was directly used for cluster generation and sequencing analysis using an Illumina Genome Analyzer according to the manufacturer’s instructions. The image files generated by the sequencer were then processed to produce digital data. The subsequent procedures included summarising the data produced, evaluating the sequencing quality and depth, calculating the length distribution of the small RNAs, and filtering contaminated reads.

### The full-length cDNA of the *Salmonella* primary Sal-1

The full-length cDNAs of the *Salmonella* primary Sal-1 were obtained using the SMARTer^TM^ RACE cDNA amplification kit (Clontech, Mountain View, CA) according to the manufacturer’s instructions. Because the RNA template was from a prokaryotic organism and lacked a polyadenylated tail, we thus added a poly(A) tail using Poly(A) Polymerase (Takara). Then, the first-strand cDNA was synthesised using a modified oligo(dT) primer (5′-RACE CDS primer A or 3′-RACE CDS primer A), and the SMARTer^TM^ II A oligonucleotide was added for 5′-RACE cDNA synthesis. The gene-specific antisense primer sal-GSP1 was used for 5′-RACE amplification, and sal-GSP2 was used for 3′-RACE amplification. The cDNA was amplified using the Advantage 2 PCR kit (Clontech) with the above gene-specific primers and the Universal Primer A Mix in the SMARTer^TM^ RACE kit. The RACE product was electrophoresed, purified, and ligated into the pMD19-T Easy vector (Takara). The ligations were transformed into *E. coli* Top 10 chemically competent cells. The plasmids were extracted from bacterial cell suspensions using the E.Z.N.A.® Plasmid Mini Kit (Omega Bio-Tek, Norcross, GA) for sequence analysis.

### Cell transfection

HT-29 cells or RAW264.7 macrophages were seeded in 6-well plates or 60-mm dishes and transfected using Lipofectamine^TM^ 2000 (Invitrogen, Carlsbad, CA) according to the manufacturer’s instructions. To knock down Dicer or AGO2, their respective active siRNAs or a scrambled negative control was used. The sequences of the siRNAs used in this study were listed in Supplementary Table S5. To overexpress Sal-1 or Pre-Sal-1 plasmids or a scrambled negative control were used. To transfect Pri-Sal-1, *in vitro* transcript primary Pri-Sal-1 RNAs were used. At 6 h post-transfection, the media was changed to DMEM supplemented with 1% FBS and the cells were harvested 48 h post- transfection.

### Northern blot analysis

Northern blot analysis was performed on Sal-1 and total RNAs isolated from *Salmonella* strain SE2472, *Salmonella*-infected cells, and Pri-Sal-1- and Pre-Sal-1-transfected cells. Total RNA was dissolved in 2x RNA loading buffer (Takara, Dalian, China), heated at 95 °C for 5 min, loaded onto denaturing 15% TBE-urea PAGE gels, transferred onto nylon membranes, and UV-cross linked with 1200mJ of energy. For northern blot analysis, an LNA probe was labelled with the non-radioactive DIG using the End Tailing Kit (Roche, Indianapolis, IN). Pre-hybridisation and hybridisation were performed according to the manufacturer’s instructions. A DIG-labelled U6 snRNA probe was used as a control. Probe detection was performed using the DIG Luminescent Detection Kit. Briefly, the blots were incubated in blocking solution for 30 min and then in anti-DIG-AP antibody solution for 30 min, followed by two washes. After equilibration, the blots were incubated with the chemiluminescent substrate CDP-Star and exposed to Kodak X-OMAT BT film.

### Western blot

The cells were rinsed with PBS and lysed in cold RIPA buffer (10 mM Tris-HCl, 1 mM EDTA, 1% sodium dodecyl sulphate, 1 mM DTT, 0.1 mM PMSF, protease inhibitors, 1% Nonidet P-40, pH 8.0). Lysates were centrifuged at 16,100 × *g* for 10 min at 4 °C to remove cell debris, and the protein content was analysed using a Micro BCA Protein Assay kit (Pierce, Rockford, IL). The proteins (40 μg) were separated by SDS-PAGE and electrotransferred to polyvinylidene difluoride (PVDF) membranes (Roche, Indianapolis, IN). The membranes were blocked with 5% non-fat dry milk or bovine serum albumin (BSA) in TBST (Tris-buffered saline plus 0.1% Tween-20) for 1 h. The blots were then probed with primary antibodies against Dicer, AGO2 or GAPDH, followed by horseradish peroxidase-conjugated secondary antibodies. Chemiluminescence detection was performed by using the ECL kit (Pierce) or exposure to X-ray film (Kodak).

### Detection of Sal-1 and Pre-Sal-1 in AGO2-associated complex

*Salmonella*-infected or Pre-Sal-1-transfected cells were lysed with lysis buffer (20 mM Tris-HCl, 150 mM NaCl, 0.5% Nonidet P-40, 2 mM EDTA, 0.5 mM DTT, 1 mM NaF, 1 mM PMSF, DNase and RNase inhibitors, and 1% Protease Inhibitor Cocktail, pH7.5) for 30 min on ice. The lysates were cleared by centrifugation (16,000 × *g*) for 10 min at 4 °C and then immunoprecipitated with mouse monoclonal anti-AGO2 antibody or mouse normal IgG followed by protein G-agarose beads at 4 °C overnight with shaking. After elution from the beads, the RNA was prepared using TRIzol LS reagent. A rabbit polyclonal anti-AGO2 antibody was used for western blot analysis. After purification, immunoprecipitated RNA was analysed by real-time RT-PCR for Sal-1 using TaqMan Sal-1 probes (Applied Biosystems) according to the manufacturer’s instructions or by semi-quantitative RT-PCR using primers (listed in Supplementary Table S5) specific for Pre-Sal-1. The PCR amplification product was collected and inserted into T-vector for sequencing.

### Construction of a *Salmonella* mutant with deletion of Sal-1 sequence

A Sal-1 deletion (ΔSal-1) mutant of SE2472 was constructed using the one-step mutagenesis method as described previously^32^. Different primers were used to amplify drug resistance genes (such as Kan^r^, Chl^r^, Spc^r^, etc.) from different plasmids (such as pKD3, pKD3, pfw5, etc.) replacing the Sal-1 sequence. And plasmid pcp20 was used to remove resistance genes.

### *In vitro* transcription of primary Pri-Sal-1

Primary Pri-Sal-1 RNAs were obtained using Riboprobe® *in vitro* Transcription Systems (Promega) according to the manufacturer’s instructions. The primers were designed for the amplification of primary Pri-Sal-1 from *Salmonella*-infected HT-29 cells according to the sequences obtained from 3′- and 5′- RACE amplification. RT-PCR amplification products were inserted into the *Eco*RI and *Hin*dIII double-digested pGEM®−11Zf( + ) (Promega) vector and sequenced. These recombinant plasmids were then linearized by digestion with *Hin*dIII (used for T7 transcription). The template DNA was purified by phenol/chloroform extraction and used for *in vitro* transcription. The DNA template was removed by digestion with DNase I following the transcription reaction.

### *In vivo* studies

Animal maintenance and experimental procedures were performed in accordance with the National Institutes of Health Guidelines for the Use of Experimental Animals and approved by the Animal Care Committee of Nanjing University (Nanjing, China). 60 female BALB/c mice (6–8 weeks old) were randomly divided into 6 groups, and one group was inoculated with PBS as a Mock infection. Briefly, 0.5–2 × 10^8^ TU of lentivirus was slowly administered into the lumens of the mouse colons via a catheter inserted 4 cm into the colon through the anus. The mice were inoculated intragastrically with 5 × 10^6^ CFU of strain SE2472 per mouse. The mice were monitored during the course of infection, and the clinical evaluation was performed according to a clinical scoring system (based on the activity of the mice, their feeding, appearance of their fur, hunched positioning, and hemorrhagic spots on the tail) as previously described with minor modifications^33^. The mice were euthanized at 3 days after inoculation, and their colons were collected to analyse the Sal-1 expression and the surviving *Salmonella* colonies. Aliquots of mouse colon tissue homogenates were used to determine the CFU/ml through serial dilution and plating onto CHROMagar^TM^ plates. The plates were incubated at 37 °C for 18 h. The number of *Salmonella* colonies was counted the next day. Each sample was analysed in triplicate, and the analysis was repeated at least twice. The CFU per sample was expressed as the average of the counts obtained. The concentrations of bacteria were recorded as CFU per mg of organ.

### Statistical analysis

Western blot and qRT-PCR results were representative of at least three independent experiments. The data are presented as the mean ± SEM of at least three independent experiments, and the differences were considered to be statistically significant at *P* < 0.05 using non-parametric tests or one-way ANOVA.

## Electronic supplementary material

Supplementary information

## References

[1] McGhie EJ, Brawn LC, Hume PJ, Humphreys D, Koronakis V (2009). *Salmonella* takes control: effector-driven manipulation of the host. Curr Opin Microbiol.

[2] Diacovich L, Gorvel JP (2010). Bacterial manipulation of innate immunity to promote infection. Nat Rev Microbiol.

[3] Finlay BB, McFadden G (2006). Anti-immunology: evasion of the host immune system by bacterial and viral pathogens. Cell.

[4] Bhavsar AP, Guttman JA, Finlay BB (2007). Manipulation of host-cell pathways by bacterial pathogens. Nature.

[5] Wallis TS, Galyov EE (2000). Molecular basis of *Salmonella*-induced enteritis. Mol Microbiol.

[6] Patel JC, Galan JE (2005). Manipulation of the host actin cytoskeleton by *Salmonella*–all in the name of entry. Curr Opin Microbiol.

[7] McGourty K (2012). *Salmonella* inhibits retrograde trafficking of mannose-6-phosphate receptors and lysosome function. Science.

[8] Roy MF, Malo D (2002). Genetic regulation of host responses to *Salmonella* infection in mice. Genes Immun.

[9] Schoen C (2005). Bacterial delivery of functional messenger RNA to mammalian cells. Cell Microbiol.

[10] Underhill DM, Goodridge HS (2012). Information processing during phagocytosis. Nat Rev Immunol.

[11] Sander LE (2011). Detection of prokaryotic mRNA signifies microbial viability and promotes immunity. Nature.

[12] Gong H (2011). A *Salmonella* small non-coding RNA facilitates bacterial invasion and intracellular replication by modulating the expression of virulence factors. PLoS Pathog.

[13] Padalon-Brauch G (2008). Small RNAs encoded within genetic islands of *Salmonella typhimurium* show host-induced expression and role in virulence. Nucleic Acids Res.

[14] Vogel J (2009). A rough guide to the non-coding RNA world of *Salmonella*. Mol Microbiol.

[15] Hudson DL (2007). Inhibition of type III secretion in *Salmonella enterica* serovar Typhimurium by small-molecule inhibitors. Antimicrob Agents Chemother.

[16] Carthew RW, Sontheimer EJ (2009). Origins and Mechanisms of miRNAs and siRNAs. Cell.

[17] Cheloufi S, Dos Santos CO, Chong MM, Hannon GJ (2010). A dicer-independent miRNA biogenesis pathway that requires Ago catalysis. Nature.

[18] Diederichs S, Haber DA (2007). Dual role for argonautes in microRNA processing and posttranscriptional regulation of microRNA expression. Cell.

[19] Cifuentes D (2010). A novel miRNA processing pathway independent of Dicer requires Argonaute2 catalytic activity. Science.

[20] Yang JS (2010). Conserved vertebrate mir-451 provides a platform for Dicer-independent, Ago2-mediated microRNA biogenesis. Proc Natl Acad Sci USA.

[21] Fraser AG (2000). Functional genomic analysis of *C. elegans* chromosome I by systematic RNA interference. Nature.

[22] Xiang S, Fruehauf J, Li CJ (2006). Short hairpin RNA-expressing bacteria elicit RNA interference in mammals. Nat Biotechnol.

[23] Gripenland J (2010). RNAs: regulators of bacterial virulence. Nat Rev Microbiol.

[24] Sorek R, Cossart P (2010). Prokaryotic transcriptomics: a new view on regulation, physiology and pathogenicity. Nat Rev Genet.

[25] Liu H (2012). *Escherichia coli* noncoding RNAs can affect gene expression and physiology of Caenorhabditis elegans. Nat Commun.

[26] Weiberg A (2013). Fungal small RNAs suppress plant immunity by hijacking host RNA interference pathways. Science.

[27] Das K, Garnica O, Dhandayuthapani S (2016). Modulation of Host miRNAs by Intracellular Bacterial Pathogens. Frontiers in cellular and infection microbiology.

[28] Verma S (2015). Salmonella Engages Host MicroRNAs To Modulate SUMOylation: a New Arsenal for Intracellular Survival. Molecular and cellular biology.

[29] Diederichs S (2008). Coexpression of Argonaute-2 enhances RNA interference toward perfect match binding sites. Proc Natl Acad Sci USA.

[30] Lu S, Manges AR, Xu Y, Fang FC, Riley LW (1999). Analysis of virulence of clinical isolates of *Salmonella enteritidis in vivo* and *in vitro*. Infection and immunity.

[31] Chen X (2008). Characterization of microRNAs in serum: a novel class of biomarkers for diagnosis of cancer and other diseases. Cell Res.

[32] Datsenko KA, Wanner BL (2000). One-step inactivation of chromosomal genes in *Escherichia coli* K-12 using PCR products. Proc Natl Acad Sci USA.

[33] Ozkaya H (2012). *Salmonella typhimurium* infections in BALB/c mice: a comparison of tissue bioluminescence, tissue cultures and mice clinical scores. New Microbiol.

