# Supplementary material for: Salmonella produce microRNA-like RNA fragment Sal-1 in the infected cells to facilitate intracellular survival
Source: Sci Rep. 2017 May 24;7:2392. doi: 10.1038/s41598-017-02669-1 (PMC5443790; doi:10.1038/s41598-017-02669-1)
Supplement: Supplementary file 1 — Supplementary information [file 41598_2017_2669_MOESM1_ESM.pdf]

Salmonella produce microRNA-like RNA fragment Sal-1 in the infected cells to facilitate intracellular survival

Hongwei Gu[1,2*], Chihao Zhao[1,2*], Tianfu Zhang[1,2*], Hongwei Liang[1,2], Xiao-Ming Wang[1], Yi Pan[1,2], Xi Chen[1,2], Quan Zhao[1,2], Donghai Li[1,2], Fenyong Liu[3†], Chen-Yu Zhang[1,2†], and Ke Zen[1,2†]

[1]State Key Laboratory of Pharmaceutical Biotechnology, Nanjing Advanced Institute for Life Sciences, School of Life Sciences, Nanjing University, Nanjing, Jiangsu 210046; [2]Jiangsu Engineering Research Center for MicroRNA Biology and Biotechnology, Nanjing, Jiangsu 210093, China; [3]School of Public Health, University of California at Berkeley, Berkeley, CA 94720, USA.

Running title: *Salmonella* produce miRNA-like Sal-1

Keywords: *Salmonella;* non-coding RNA; AGO2; infection; miRNA

* To whom correspondence should be addressed:

†Correspondences:

Ke Zen, PhD

Email: kzen@nju.edu.cn

Chen-Yu Zhang, PhD, MD

Email: cyzhang@nju.edu.cn

Fenyong Liu, PhD

Email: liu_fy@berkeley.edu

Supplementary Table S1. Sequences and reads of miRNA-like RNA fragments in *salmonella*-infected intestinal epithelial cells detected by Solexa (Top 10).

**No.**	**Name**	**Sequence***	**Reads**
1	Sal-1	TGTGGGCACTCGAAGATACGGATT	1852
2	Sal-2	ATGCGAGAGTAGGGAACTGCCAGGCAT	550
3	Sal-3	TCCTCTGTAGTTCAGTCGGTAGAACGGC	491
4	Sal-4	GAAGGTGGCGGAATTGGTAGACG	397
5	Sal-5	GCCCGGATGGTGGAATCGGTA	262
6	Sal-6	TTCAGTCGGTAGAACGGCG	170
7	Sal-7	AGGGGCGTAGTTCAATTGGT	155
8	Sal-8	GAGTAGGGAACTGCCAGGCAT	137
9	Sal-9	ACCTGTGTGACTGCGTACCTT	129
10	Sal-10	GCGGGCATCGTATAATGGCTAT	117
60	miR-107	AGCAGCATTGTACAGGGCTATCA	1569

Supplementary Table S2. Characterization of Pri-Sal-1 (A) in bacterial species.

Species	Locus	Identities	Strand (plus/minus)
S. Enteritidis (str. P125109)	293906-294306	397/402(99%)	P
	3453908-3453510	393/402(98%)	M
	4266596-4266994	394/402(98%)	P
S. Dublin (str. CT_02021853)	289730-290130	396/402(99%)	P
	3613091-3612693	394/402(98%)	M
	4424832-4425232	398/402(99%)	P
S. Cubana (str. CFSAN002050)	453066-453466	397/402(99%)	P
	1301343-1301741	390/402(97%)	P
	4628790-4628391	391/402(97%)	M
S. Heidelberg (str. 41578)	510190-509790	397/402(99%)	M
	1354868-1355266	389/402(97%)	P
	2089538-2089938	388/402(97%)	P
S. Newport (str. SL254)	291068-291468	397/402(99%)	P
	3554309-3553911	388/402(97%)	M
	4384533-4384931	394/402(98%)	P
S. Javiana (str. CFSAN001992)	2631389-2630989	396/402(99%)	M
	3429360-3429760	396/402(99%)	P
	1935691-1935291	392/402(98%)	M
S. Gallinarum/pullorum (str. RKS5078)	290858-291258	396/402(99%)	P
	4224641-4225041	396/402(99%)	P
	3416721-3416321	395/402(98%)	M
S. Paratyphi A (str. ATCC 9150)	293036-293436	396/402(99%)	P
	4166698-4167098	395/402(98%)	P
	3378679-3378279	391/402(97%)	M
S. Paratyphi B (str. SPB7)	289793-290193	395/402(98%)	P
	3530926-3530526	391/402(97%)	M
	4335253-4335649	385/402(96%)	P
*S.*Paratyphi C (str. RKS4594)	285201-285604	396/402(99%)	P
	3505212-3504812	395/402(98%)	M
	4074422-4074823	389/402(96%)	P
S. Schwarzengrund (str. CVM19633)	291315 -291715	390/402(97%)	P
	3470206-3469806	393/402(98%)	M
	4260397-4260797	395/402(98%)	P
S. Typhimurium (str. LT2)	288896-289294	393/402(98%)	P
	3572292-3571892	394/402(98%)	M
	4394392-4394790	389/402(97%)	P
S. Typhi (str. CT18)	287194-287594	392/402(98%)	P
	3558138-3557737	392/402(98%)	M
	4257207-4257607	392/402(98%)	P
E. coli O157:H7 (str. TW14359)	5075840-5076241	394/402(98%)	P
	226796-227198	394/402(98%)	P
Shigella flexneri 2002017	4266481-4266880	392/402(98%)	P
	213868-214269	390/402(97%)	P

Supplementary Table S3. Characterization of Pri-Sal-1 (B) in bacterial species.

Species	Locus	Identities	Strand (plus/minus)
S. Enteritidis (str. P125109)	294014-294306	288/291(98%)	P
	2754007-2753715	287/291(98%)	M
	3453800-3453510	289/291(99%)	M
	4266704-4266994	290/291(99%)	P
S. Dublin (str. CT_02021853)	289838-290130	287/291(98%)	P
	3612983-3612693	290/291(99%)	M
	2880785-2880493	287/291(98%)	M
	4424940-4425232	289/291(99%)	P
S. Cubana (str. CFSAN002050)	453174-453466	289/291(99%)	P
	3801665-3801375	289/291(99%)	M
	1301451-1301741	286/291(98%)	P
	4628683-4628391	283/291(97%)	M
S. Heidelberg (str. 41578)	510082-509790	288/291(98%)	M
	4558553-4558261	287/291(98%)	M
	1354976-1355266	285/291(98%)	P
	2089646-2089938	280/291(96%)	P
S. Newport (str. SL254)	4384641-4384931	288/291(99%)	P
	291176-291468	288/291(98%)	P
	2804362-2804070	288/291(98%)	M
	3554201-3553911	285/291(98%)	M
S. Javiana (str. CFSAN001992)	2631281-2630989	287/291(98%)	M
	3429468-3429760	287/291(98%)	P
	4197999-4198291	287/291(98%)	P
	1935583-1935291	284/291(97%)	M
S. Gallinarum/pullorum (str. RKS5078)	290966-291258	287/291(98%)	P
	2731361-2731069	287/291(98%)	M
	3416613-3416321	287/291(98%)	M
	3460045-3459753	287/291(98%)	M
	3554498-3554206	287/291(98%)	M
	3713203-3712911	287/291(98%)	M
	4224749-4225041	287/291(98%)	P
S. Paratyphi A (str. ATCC 9150)	293144-293436	287/291(98%)	P
	4166806-4167098	287/291(98%)	P
	3874980-3875270	285/291(98%)	P
	3974113-3974403	285/291(98%)	P
	2619682-2619391	284/291(97%)	M
	3378571-3378279	283/291(97%)	M
S. Paratyphi B (str. SPB7)	289901-290193	286/291(98%)	P
	2815622-2815330	283/291(97%)	M
	3530818-3530526	283/291(97%)	M
	4335361-4335649	281/291(97%)	P
*S.*Paratyphi C (str. RKS4594)	285312-285604	288/291(98%)	P
	2800941-2800649	288/291(98%)	M
	3505104-3504812	288/291(98%)	M
S. Schwarzengrund (str. CVM19633)	2754501-2754209	289/291(99%)	M
	4260505-4260797	287/291(98%)	P
	3470098-3469806	284/291(97%)	M
	291423-291715	282/291(96%)	P
S. Typhimurium (str. LT2)	289004-289294	289/291(99%)	P
	2801837-2801545	287/291(98%)	M
	4394500-4394790	285/291(98%)	P
	3572184-3571892	285/291(97%)	M
S. Typhi (str. CT18)	287302-287594	283/291(97%)	P
	2717717-2717425	283/291(97%)	M
	3423618-3423326	283/291(97%)	M
	3600245-3599953	283/291(97%)	M
	3749246-3748954	283/291(97%)	M
	4257315-4257607	283/291(97%)	P
	3558030-3557737	283/291(97%)	P
E. coli O157:H7 s(tr. TW14359)	226906-227198	282/291(96%)	P
	4223073-4222781	282/291(96%)	M
	4890652-4890944	282/291(96%)	P
	5034923-5035215	282/291(96%)	P
	5075949-5076241	282/291(96%)	P
Shigella sonnei 53G	3736452-3736160	283/291(97%)	M
	236143-236435	280/291(96%)	P

Supplementary Table S4. Characterization of Pri-Sal-1 (C) in bacterial species.

Species	Locus	Identities	Strand (plus/minus)
S. Enteritidis (str. P125109)	294014-294210	192/195(97%)	P
	2754007-2753811	192/195(97%)	M
	3453800-3453606	194/195(99%)	M
	4266704-4266898	195/195(100%)	P
S. Dublin (str. CT_02021853)	289838-290034	191/195(97%)	P
	3612983-3612789	195/195(100%)	M
	2880785-2880589	192/195(97%)	M
	4424940-4425136	193/195(98%)	P
S. Cubana (str. CFSAN002050)	453174-453370	193/195(98%)	P
	3801665-3801471	193/195(99%)	M
	1301451-1301645	190/195(97%)	P
	4628683-4628487	187/195(95%)	M
S. Heidelberg (str. 41578)	510082-509886	192/195(97%)	M
	4558553-4558357	191/195(97%)	M
	1354976-1355170	189/195(97%)	P
	2089646-2089842	185/195(94%)	P
S. Newport (str. SL254)	4384641-4384835	192/195(98%)	P
	291176-291372	193/195(98%)	P
	2804362-2804166	193/195(98%)	M
	3554201-3554007	190/195(97%)	M
S. Javiana (str. CFSAN001992)	2631281-2631085	191/195(97%)	M
	3429468-3429664	191/195(97%)	P
	4197999-4198195	191/195(97%)	P
	1935583-1935291	188/195(95%)	M
S. Gallinarum/pullorum (str. RKS5078)	290966-291162	191/195(97%)	P
	2731361-2731165	191/195(97%)	M
	3416613-3416417	191/195(97%)	M
	3460045-3459849	191/195(97%)	M
	3554498-3554302	191/195(97%)	M
	3713203-3713001	191/195(97%)	M
	4224749-4224945	191/195(97%)	P
S. Paratyphi A (str. ATCC 9150)	293144-293340	191/195(97%)	P
	4166806-4167002	191/195(97%)	P
	3874980-3875174	189/195(97%)	P
	3974113-3974307	189/195(97%)	P
	2619682-2619487	188/195(96%)	M
	3378571-3378375	187/195(95%)	M
S. Paratyphi B (str. SPB7)	289901-290097	191/195(97%)	P
	2815622-2815426	188/195(96%)	M
	3530818-3530622	188/195(96%)	M
	4335361-4335553	186/195(95%)	P
*S.*Paratyphi C (str. RKS4594)	285312-285508	193/195(98%)	P
	2800941-2800745	193/195(98%)	M
	3505104-3504908	193/195(98%)	M
S. Schwarzengrund (str. CVM19633)	2754501-2754305	193/195(98%)	M
	4260505-4260701	191/195(97%)	P
	3470098-3469902	188/195(96%)	M
	291423-291619	187/195(95%)	P
S. Typhimurium (str. LT2)	289004-289198	194/195(99%)	P
	2801837-2801641	191/195(97%)	M
	4394500-4394694	190/195(97%)	P
	3572184-3571988	190/191(96%)	M
S. Typhi (str. CT18)	287302-287498	187/191(95%)	P
	2717717-2717521	187/191(95%)	M
	3423618-3423422	187/191(95%)	M
	3558030-3599834	187/191(95%)	M
	3600245-3600049	187/191(95%)	M
	4257315-4257511	187/191(95%)	P
	3749246-3749050	187/191(95%)	P
E. coli O157:H7 s(tr. TW14359)	226906-227102	188/195(95%)	P
	4223073-4222877	188/195(95%)	M
	4890652-4890848	188/195(95%)	P
	5034923-5035119	188/195(95%)	P
	5075949-5076145	188/195(95%)	P
Shigella sonnei 53G	3736452-3736256	188/195(95%)	M
	236143-236339	187/195(95%)	P

Supplementary Table S5. Oligonucleotide sequence lists

No.	Name	Sequence (5’ to 3’)
1	sal-GSP1	TACGTGTTCACTCTTGAGACT
2	sal-GSP2	GCACTGCTCTTTAACAATTTATCAGAC
3	ago2-A	AAGGAUAUGCCUUCAAGCCUCdTdT
4	ago2-B	GAGGCUUGAAGGCAUAUCCUUdTdT
5	dicer-A	UGCUUGAAGCAGCUCUGGAdTdT
6	dicer-B	UCCAGAGCUGCUUCAAGCAdTdT
7	LNA probe	AATCCGTATCTTCGAGTGCCCACA
8	U6 probe	CTGCGCAAGGATGACACGCAAAT
9	Pre-Sal-1 P1	TGTGGGCACTCGAAGATAC
10	Pre-Sal-1 P2	TACGTGTTCACTCTTGAGACT

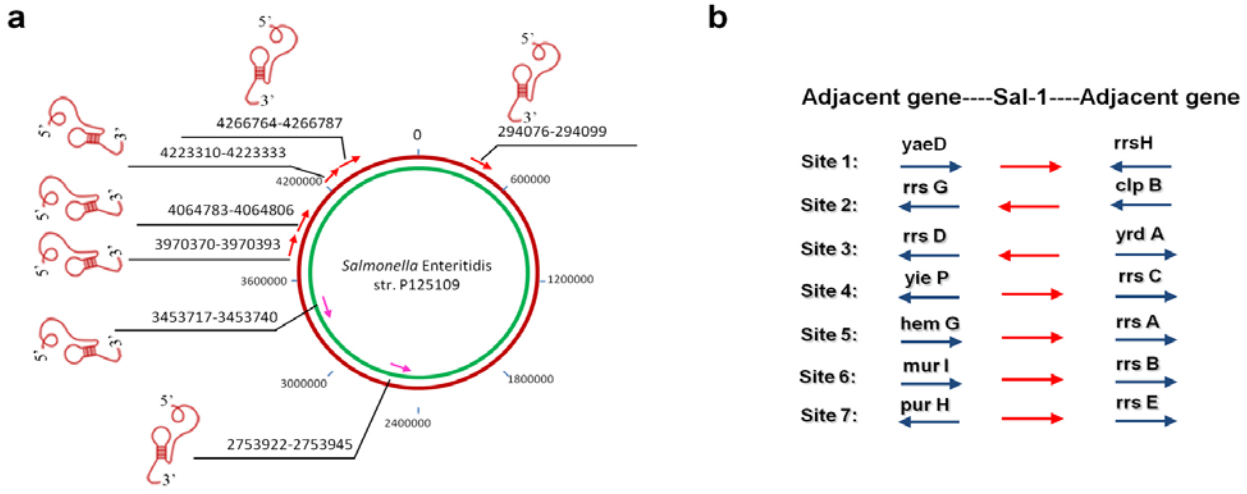

Figure S1. Multiple locations of Sal-1 sequence in *Salmonella* genome. (a) The seven sites of Sal-1 sequence in the *Salmonella* genome, referring the *Salmonella* enterica serovar Enteritidis strain P125109 genome sequence. All seven Sal-1 copies were located in the non-coding region. (b) The adjacent genes of Sal-1 sequence at seven different sites.

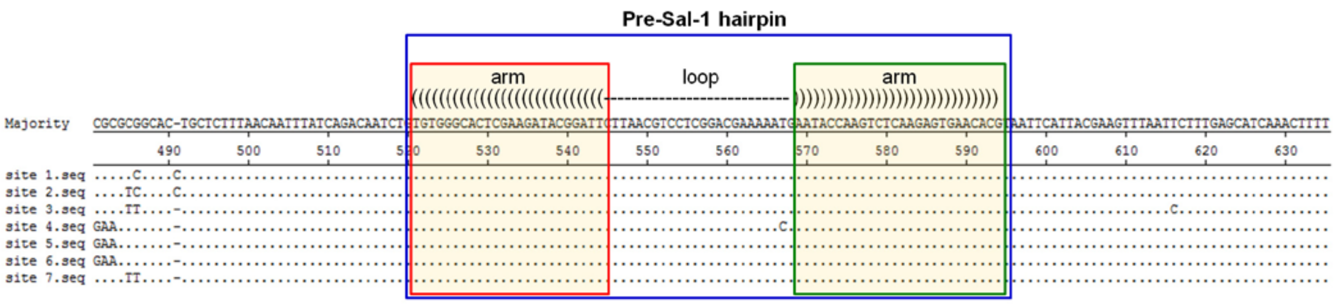

Figure S2.Sequence analysis and alignment of Sal-1 among the seven *Salmonella* non-coding regions (referring to *S. enteritidis* strain P125109). The Sal-1 sequence is marked with a red frame, and the putative Pre-Sal-1 hairpin, arm, and loop are highlighted.

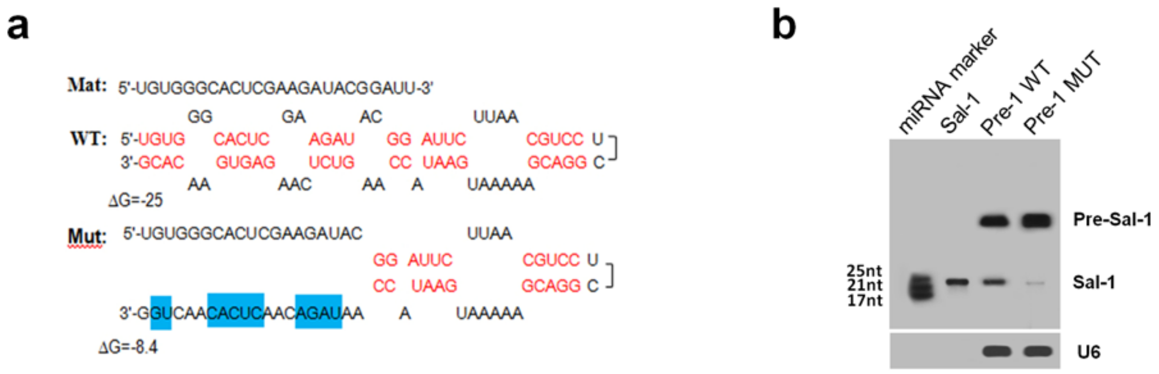

Figure S3. Sal-1 biogenesis is hairpin structure-dependent. (a) Schematic of the Pre-Sal-1 and its mutated hairpin. Site mutants were generated in the context of the Pre-Sal-1 plasmid (Pre-1). (b) Northern blot analysis of Sal-1 biogenesis. Note that Pre-Sal-1 (Pre-1) is cleaved by Ago2 into mature Sal-1, but Pre-1 (MUT) cannot be processed into mature Sal-1.

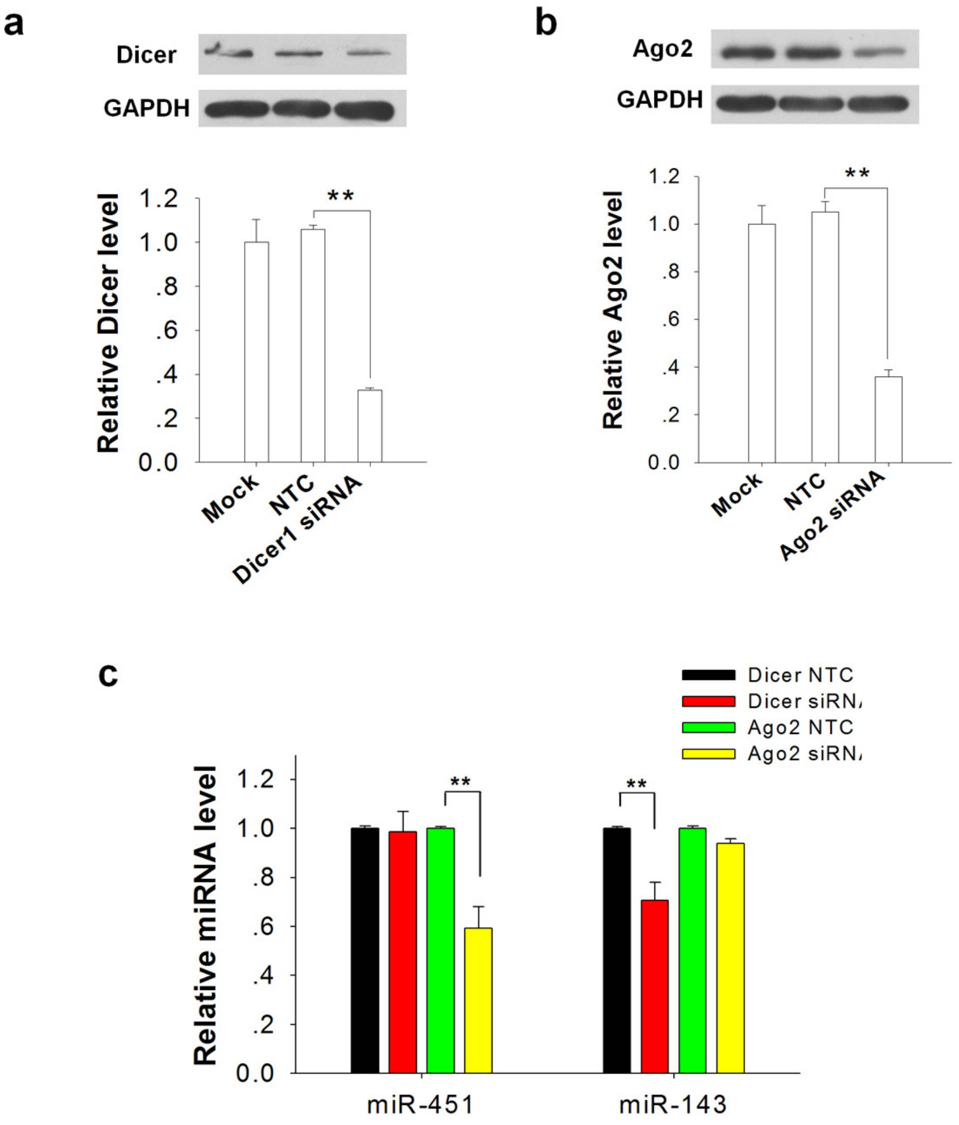

Figure S4. Biogenesis of Sal-1 in human intestinal HT-29 cells is Dicer-independent but Ago2-dependent. (a) Western blot detection of Dicer expression in HT-29 cells using Dicer-specific siRNA. (b) Ago2-specific siRNA was transfected into HT-29 cells to knockdown Ago2 expression. (c) Expression level of miR-451 and miR-143 in HT-29 cells after knocking down Dicer and Ago2 using Dicer- or Ago2-specific siRNA, respectively. The data are presented as the mean ± SEM (n=3). **, *P*<0.01.

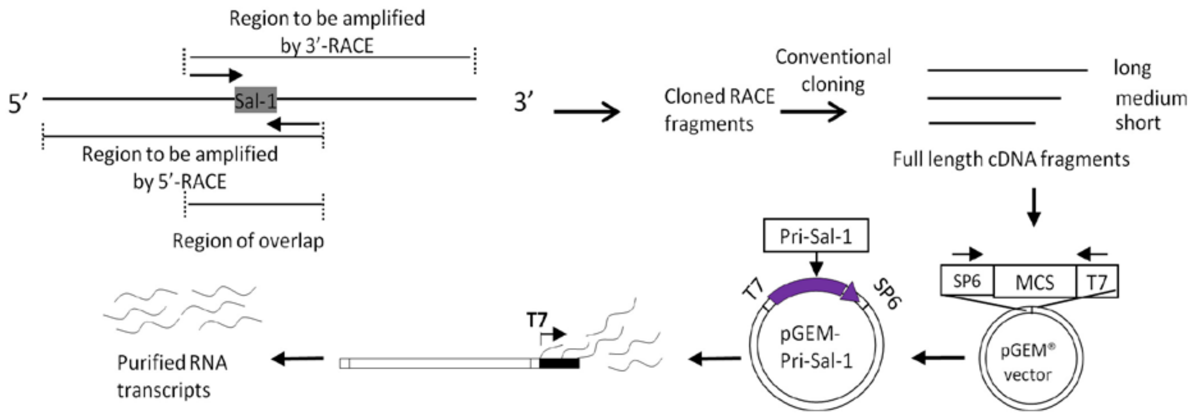

Figure S5. Diagram of 3'- and 5'-RT-RACE amplification of primary Sal-1 from infected HT-29 cells.

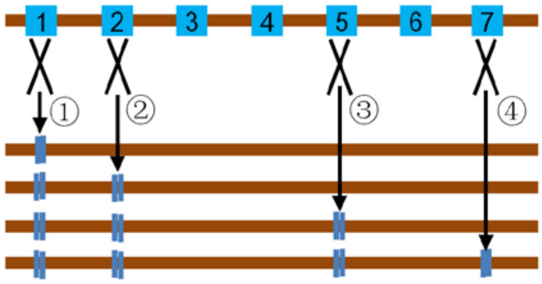

Figure S6. Depict of deletion of Sal-1 sequence at 4 locations.

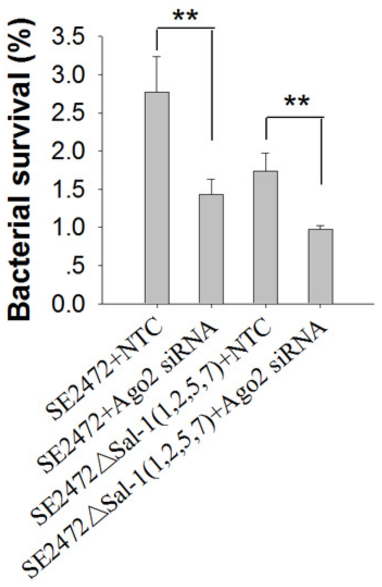

Figure S7. Bacterial survival rate in HT-29 cells infected with SE2472 or SE2472△Sal-1(1,2,5,7) with or without *Ago2* silence. **, $P<0.01$.
